# Role of Image-Free Robotic-Assisted Technology in Enhancing Accuracy of Implant Sizing for Total Knee Arthroplasty

**DOI:** 10.7759/cureus.74256

**Published:** 2024-11-22

**Authors:** Sanjay B Londhe, Kunal Patel, GovindKumar Baranwal, Ashit Shah, Dhruv Shah

**Affiliations:** 1 Department of Orthopaedics, Criticare Asia Hospital, Mumbai, IND; 2 Orthopaedics, Namah Hospital, Mumbai, IND

**Keywords:** end-stage osteoarthritis, femoral component prediction, image-free robotic-assisted technology, implant sizing accuracy, surgical precision, total knee arthroplasty (tka)

## Abstract

Background

The selection of properly sized implants is essential to achieve a well-balanced knee and favorable clinical outcomes following Total Knee Arthroplasty (TKA). There is limited evidence in the literature regarding the effectiveness of robotic-assisted technology (RA-TKA) without imaging in accurately predicting implant sizes. Our aim was to provide an evidence-based assessment of this technology’s accuracy in selecting appropriate implant sizes during robotic-assisted, image-free TKA.

Methods

This study included 50 consecutive patients who underwent image-free robotic-assisted primary TKA for end-stage knee osteoarthritis, excluding those with prior knee surgeries or undergoing revision TKA. The same surgical team performed all procedures using a medial parapatellar approach and an image-free robotic system with a handheld saw. Initial data from the first 10 cases showed 100% accuracy in implant size prediction. We calculated a sample size of 28 patients to achieve a 90% reduction in prediction error. Femoral registration points were marked (femur center, Whiteside’s line, distal medial condyle, distal lateral condyle, posterior medial condyle, posterior lateral condyle, and anterior femur cortex), and implant sizing suggested by the robotic system was verified against trial components by an independent observer. Efficacy was compared with historical controls using the Chi-square test, with significance set at p<0.05.

Results

The image-free robotic system had an accuracy of 92% (46 out of 50) in predicting the exact femoral component and 100% (50 out of 50) accuracy in predicting ±1 size femur component. Compared to the historical control, the accuracy of implant size prediction using the image-free robotic system was statistically significant (Chi-square test, p = 0.0005 and p = 0.0021, respectively).

Conclusion

The findings from this study highlight the effectiveness of image-free robotic-assisted technology in achieving precise implant sizing during primary TKA for end-stage osteoarthritis over traditional approaches. The system’s improved accuracy in femoral sizing suggests a potential shift toward more reliable, data-driven implant selection that could minimize intraoperative adjustments. By promoting a standardized approach to component fitting, image-free robotics may help optimize surgical consistency, thereby supporting better long-term implant performance and patient satisfaction. These results encourage further exploration into image-free robotic systems as a valuable tool in advancing knee arthroplasty outcomes.

## Introduction

Total knee arthroplasty (TKA), a “gold standard” surgical procedure for the management of end-stage arthritis of the knee joint, contributes to improved life quality and effective pain relief [1]. Still, almost 10-20% of TKA patients remain dissatisfied [1,2]. The basic principles involved in the TKA procedure are to achieve acceptable limb alignment, have well-balanced flexion and extension gaps on the medial and lateral sides, and use accurate-sized implants. This helps in improving the patient-reported clinical outcome measures (PROMS) and implant longevity. The use of accurate-sized implants is critically important in achieving this outcome.

To achieve the desired limb alignment, as well as equal flexion and extension gap balance or accurate rotation of femoral and tibial components, the surgeon may need to select slightly undersized or oversized components in the sagittal or coronal plane [3,4]. Coronal plane component overhang may lead to undesirable outcomes like irritation of soft tissue, increased postoperative bleeding, impingement of the popliteus tendon, and increased post-TKA pain [5,6]. Coronal plane undersized components may increase the chances of implant subsidence, osteolysis, and premature implant loosening [7,8]. Sagittal plane femur component overhang results in patella-femoral joint overstuffing and increased pressures, resulting in decreased post-TKA knee flexion and increased chances of anterior knee pain. Undersized sagittal plane femur component may lead to femur anterior cortex notching. Both oversized and undersized sagittal plane femur components result in posterior offset deviation and asymmetry of the flexion gap [9].

Any method that helps to predict the accurate size of the implants will help minimize the problems mentioned above associated with oversizing or undersizing the TKA components. This has the potential to improve the PROMS and to avoid the need for revision surgery [10,11]. In the past, manual and digital templating was employed on the pre-operative radiographs of the knee to predict accurate TKA implant sizes. These methods are not very useful as they showed very low accuracy, i.e., 42.5%-83.5% for the femur component and 48.5%-90% for the tibia component [12-14]. Pre-operative patients’ characteristics like gender, weight, and height have been found to have low accuracy in predicting the accurate TKA implant sizes [15]. The use of pre-operative computerized tomography or magnetic resonance imaging for implant templating or patient-specific instrumentation has also shown low accuracy in predicting accurate implant sizes [16,17]. Computer-assisted navigation TKA surgery has also been shown to have femur component oversizing by one or two sizes due to anterior bowing of the femur bone, especially in females [18,19].

The advent of robotic-assisted (RA) TKA offers the potential to accurately predict appropriate implant sizes for TKA. A preoperative 3D CT scan has been shown to accurately predict the femur and tibia implant sizes, with accuracy reaching 100% when one size smaller or larger implant is included [20-22]. Limited information exists on the effectiveness of image-free handheld robotic systems in accurately predicting TKA implant sizes. A study by Krishna Kiran et al. [23] using the Navio handheld robotic system (Navio, Smith & Nephew, Memphis, TN, USA) reported an accuracy of 63% for exact femoral component size prediction, 94% for ±1 size, and 99.4% for ±2 sizes. The same study showed 15.8%, 55.8%, and 76.4% accuracy in predicting exact, ±1 and ±2 tibia component sizes. The aim of the current study is to assess the efficacy of the image-free robotic system in determining the correct implant sizes while performing image-free RA-TKA. The accurate prediction of implant size helps the surgeon to use the implant best suited for the individual patient’s anatomy, which will have the potential to improve the post-TKA clinical recovery and PROMS.

## Materials and methods

It is a single-center retrospective assessment of data obtained from the prospectively enrolled patients operated by the same surgical team who underwent RA-TKA with an image-free Velys (Johnson and Johnson Medtech, New Jersey, USA) robotic system. The patients who underwent image-free primary robotic-assisted TKA for end-stage knee osteoarthritis were identified, and data was collected in the electronic case report form. Patients who were undergoing revision TKA or had a history of prior knee surgery were excluded. Ethics committee approval was not needed as this was a retrospective observational study. Informed consent was obtained from all the patients prior to surgery. Preliminary findings of the first 10 cases indicated 100% accuracy of the robotic system in accurately predicting implant sizes. The estimated sample size required was 28 patients to achieve a 90% reduction in implant size prediction error, with an alpha error of 0.05, a beta error of 0.20, and a study power of 80%, based on an assumed 30% error rate in femoral implant sizing from historical control data. All the patients underwent RA-TKA with an image-free hand-held robotic system and were implanted with a Depuy Attune® Knee (Depuy, Johnson & Johnson Medtech, New Jersey, USA). The same surgical team performed all the RA-TKA procedures. A tourniquet was used in all patients.

Surgical plan

**Figure 1:**
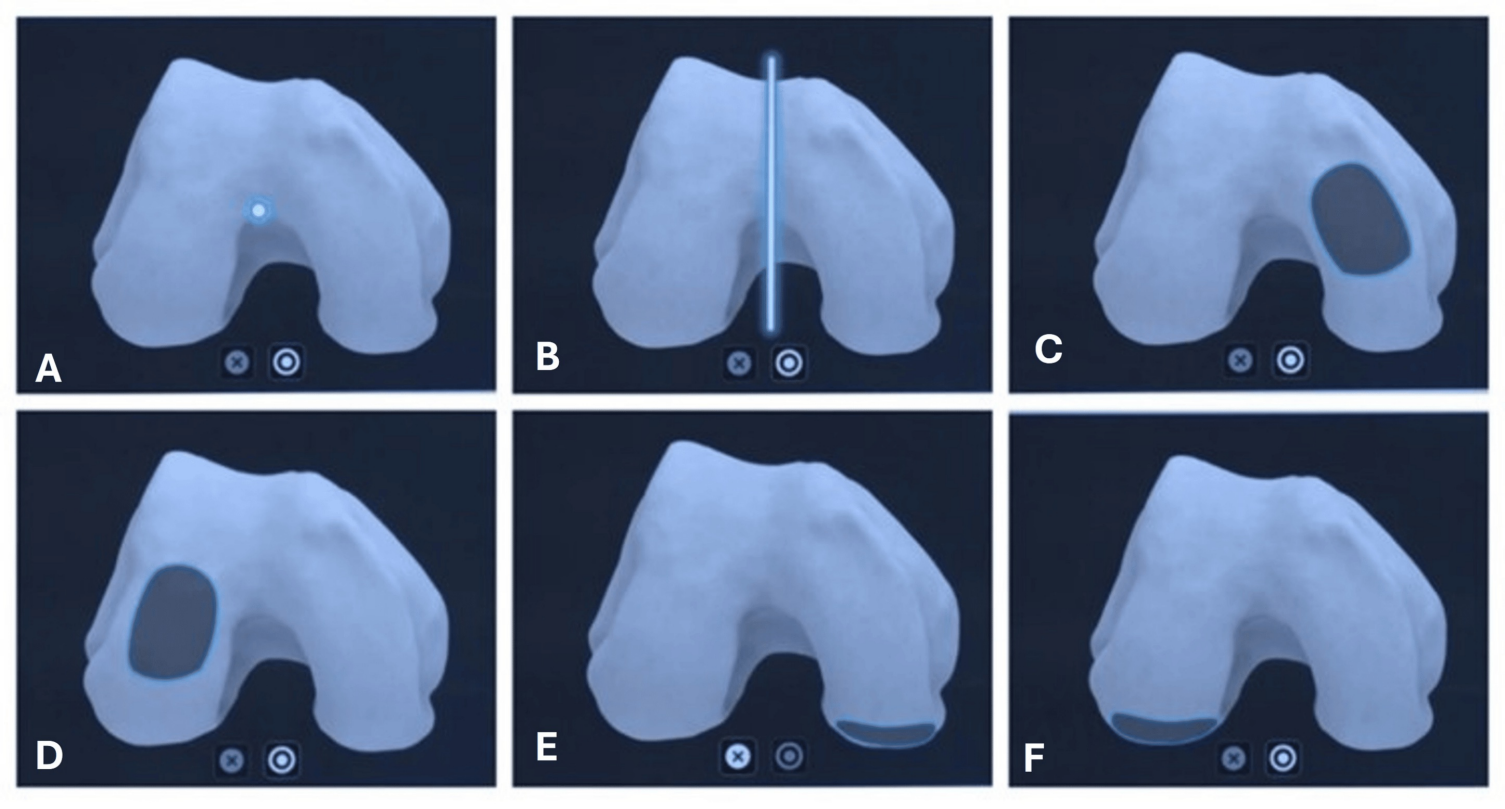
Anatomic landmark points of femur. Image Credit (Corresponding author): Dr. Sanjay B. Londhe

A medial parapatellar arthrotomy approach was used (which is the same used in previous clinical studies used for historical control). Upon doing the medial arthrotomy, osteophytes from the femur and tibia were removed. The femur and tibia pins were inserted (two each) within the surgical incision. The femoral and tibial tower trackers were attached to these femur and tibial pins. Following this step, the bony landmarks, including the hip, knee, and ankle (HKA) center, were registered. 3D mapping of the femur and the proximal tibia was done with the help of a pointer probe. During the TKA procedure, the following registration points were marked on the femur: 1) femur center, 2) Whiteside’s line, 3) distal medial condyle, 4) distal lateral condyle, 5) posterior medial condyle, 6) posterior lateral condyle, and 7) anterior femur cortex (Figure 1).

**Figure 2:**
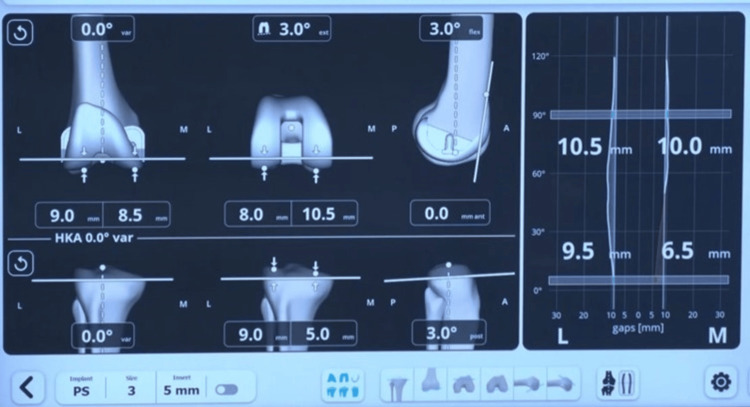
Manufacturer’s software showing femur implant size and tentative operative plan. Image Credit (Corresponding author): Dr. Sanjay B. Londhe

Upon registration of these points, the robotic system software suggested the likely femur implant size (manufacturer’s plan) (Figure 2).

**Figure 3:**
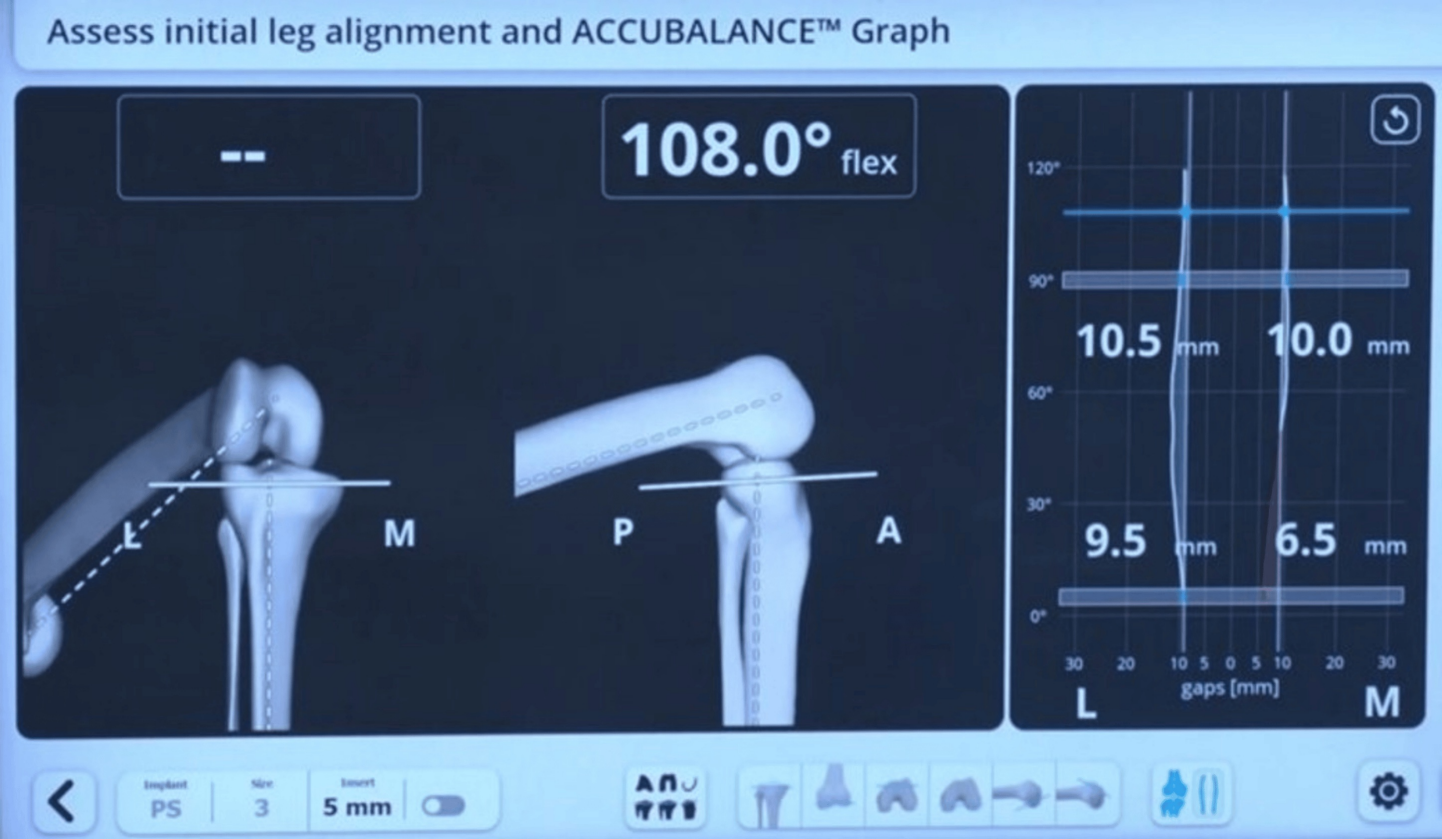
Pre-adjustment captured gaps in 0-30-60-90 degrees of flexion. Image Credit (Corresponding author): Dr. Sanjay B. Londhe

The distal femur resection was planned to be perpendicular to the femur's mechanical axis, at a level that corresponded to the amount of bone resection necessary to accommodate the selected femoral implant. The smallest possible femur implant restoring the posterior offset without leading to notching of the anterior cortex of the femur is chosen. By default, the manufacturer’s plan kept the femur component flexion at 3 degrees and external rotation at 3 degrees of external rotation (Figure 2). The upper tibial resection was carried out, removing 9 mm from the unaffected tibial condyle. Then a sensor tensor was inserted between the resected upper tibial surface and the femur. The knee was moved through a range of motion (ROM), and the medial and lateral gap values are captured from full extension through 30-60-90 degrees and maximum flexion of the knee (Figure 3).

**Figure 4:**
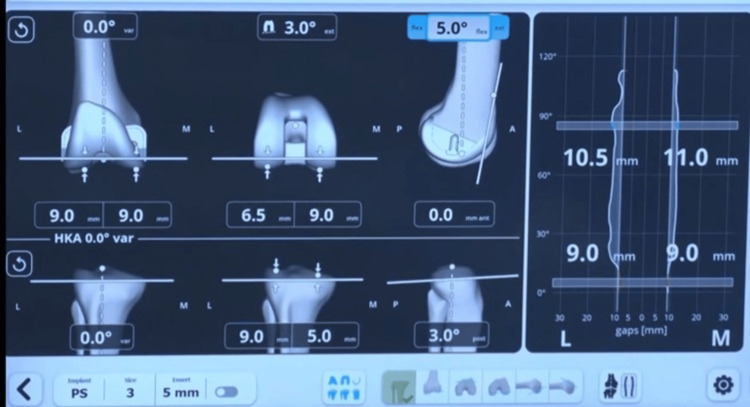
Post-adjustment captured gaps in full extension to full flexion. Image Credit (Corresponding author): Dr. Sanjay B. Londhe

At this stage, the operating surgeon adjusted various femoral implant parameters, including proximal or distal positioning of the femur cut, setting the distal femur cut at 1°-3° varus or valgus, internal or external rotation of the femoral component, altering the flexion angle of the femoral component, and shifting the component anteriorly or posteriorly (Figure 4).

This approach ensures a well-balanced knee in both flexion and extension across the full range of motion (ROM). Once the surgeon is satisfied with the adjusted plan, the necessary cuts are made using the handheld robotic-assisted saw. The accuracy of these cuts was then confirmed using a handheld pointer device. The flexion and extension gap balance was checked again with the trial femur and tibia implants. If necessary, titrated soft tissue releases were performed to achieve the desired gap balance. Once satisfactory balance is achieved, the rest of the definite femur and tibia component implantation is carried out in the usual manner. 

Data analysis and interpretation

**Figure 5:**
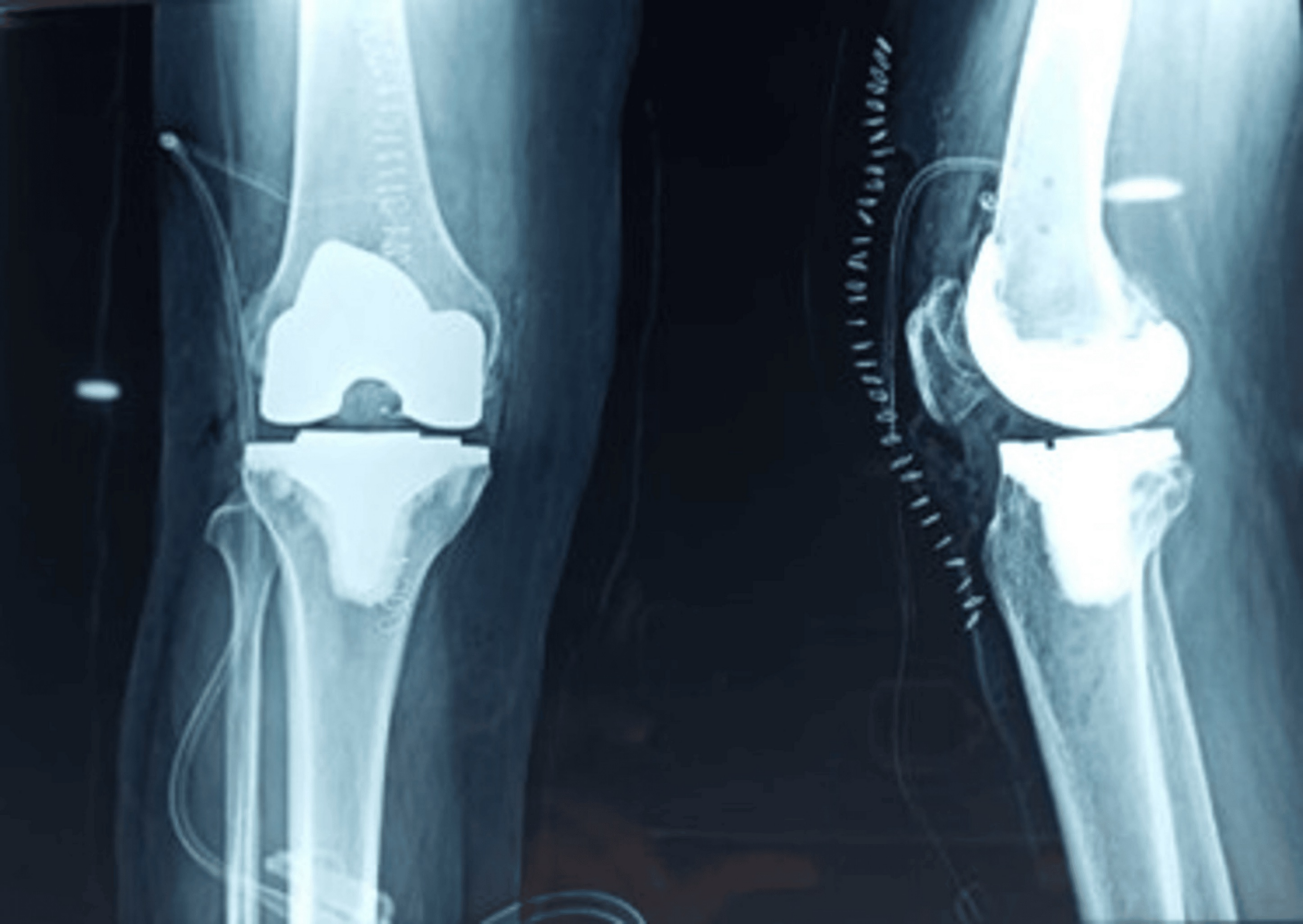
Post operative X-ray. Image Credit (Corresponding author): Dr. Sanjay B. Londhe

The femoral component size predicted by the robotic system software was documented. The final femoral implant size, based on the surgeon’s customized plan and verified using a trial component to assess mediolateral and anteroposterior fit against the patient's anatomy, was also recorded. An independent observer, who was not involved in the surgery, examined the predicted and actual implanted femoral sizes. The same observer also assessed post-operative AP and lateral radiographs (Figure 5), following radiographic markers outlined by Peek et al. [12].

Parameters examined included the presence or absence of femoral notching, any gap greater than 2 mm between the anterior femoral cortex and implant, and the restoration of the posterior femoral contour. The efficacy of the image-free robotic system in accurately predicting femoral implant size was compared to historical controls using the Chi-square test, with significance defined at p<0.05.

## Results

The study enrolled 50 consecutive patients, including 13 males and 37 females. The age range was from 43 to 87 years, with a mean of 68.5 years and a standard deviation of 8.98 (Table 1).

**Table 1:** Pre-operative patient demographics. ASA: American Society of Anesthesiologists

Patient demographics	N=50
Mean age (Years)	68.5
Gender (Male/Female)	13/ 37
Body mass index, mean, range (Kg/m^2^)	29.3 (20.2 -37.5)
Side (Right/Left)	28/ 22
ASA grade, mode, range	2 (1–3)

The image-free robotic system had an accuracy of 92% (46 out of 50) in predicting the exact femoral component and 100% (50 out of 50) accuracy in predicting ±1 size femur component. The accuracy of the image-free robotic system was statistically significant compared to the historical control (Chi-square test, p = 0.0005 and p = 0.0021) (Table 2).

**Table 2:** Comparison between RA-TKA and CTKA as regards percentage match and level of agreement between robot-predicted and implanted component size for the exact size, ± 1 size with p-value. CTKA: Conventional total knee arthroplasty

Details	Exact size	± 1 size
Imageless Robotic System	92%	100%
CTKA	62.5	82.5
P-Value	0.0005	0.0021

## Discussion

The results of our study clearly illustrate the effectiveness of the image-free robotic system in accurately determining implant sizes during image-free RA-TKA. Robotic assistance in TKA improves the precision of implant positioning and aids in selecting the appropriate femoral implant size for an optimal fit.

Accurate sizing and alignment of the femoral and tibial implants are crucial for long-term implant survival and favorable clinical outcomes. A study by Dennis et al. analyzing post-TKA pain identified implant overhang as a leading cause of post-operative pain and patient dissatisfaction [5]. The overhang of the femur/tibia implant causes impingement of the soft tissue, which in turn leads to the formation of intraarticular fibrous bands. These intra-articular fibrous bands, in turn, irritate the surrounding tendons and ligaments. Undersizing the femoral component can lead to mid-flexion instability [24] and raise the risk of notching the anterior femoral cortex, which may subsequently result in a periprosthetic femoral fracture [25].

The study by Bonin et al. [26] reported an incidence of femoral component overhang in 66% of cases and tibial component overhang in 60% of cases. This has the potential to cause poor postoperative patient-reported outcomes. Mahoney et al. [6] reported that femoral component overhang exceeding 3 mm doubles the risk of knee pain.

Pre-operative CT-based templating in RA-TKA has shown a high level of accuracy in predicting femoral and tibial implant sizes [20-22]. However, a downside to this approach is its association with radiation exposure.

There are a few limitations to our study. The primary limitation is that it was conducted at a single center, with all TKA procedures performed by the same surgical team. Nevertheless, this approach minimizes potentially confounding factors, such as intraoperative variability caused by different surgeons or changes in operating room support staff. The second limitation is that multiple patient-related factors like ethnicity, age, gender, and different TKA alignment philosophies were not considered for this study. These factors have a definitive influence on the femur implant size. Further studies are needed at multiple centers involving different surgical teams utilizing different alignment philosophies.

A key strength of our study is that, to the best of our knowledge, it is the first to evaluate the effectiveness of the image-free robotic in accurately determining femoral implant size.

## Conclusions

This study demonstrates that image-free robotic-assisted technology in primary TKA is highly effective in accurately predicting femoral implant sizes for patients with end-stage knee osteoarthritis. Using a handheld robotic system, the surgical team achieved a 92% exact match rate in femoral component size prediction, with a perfect 100% accuracy rate within ±1 size, significantly outperforming historical control data. This precision suggests that image-free robotic systems provide a substantial advantage in optimizing implant selection, which is essential for achieving a well-balanced knee across the full range of motion. The flexibility to make real-time adjustments and verify each step via robotic assistance likely contributed to the observed accuracy, as it reduced the variability commonly seen with manual techniques. This approach is particularly beneficial in reducing potential postoperative complications, such as implant overhang or under-sizing, which are known to contribute to patient dissatisfaction and increase the risk of pain. The use of real-time feedback from robotic-assisted systems provides surgeons with an opportunity to refine their operative plan on a case-by-case basis, aligning implant components with a high degree of accuracy tailored to the patient’s unique anatomy. The results underscore the efficacy of image-free robotic technology as a powerful tool for improving surgical precision and consistency in TKA, with implications for enhanced long-term outcomes. Further research involving larger multicenter cohorts is recommended to validate these findings and assess their generalizability across diverse patient populations.
